# Computational Identification of the Proteins Associated With Quorum Sensing and Biofilm Formation in *Mycobacterium tuberculosis*

**DOI:** 10.3389/fmicb.2019.03011

**Published:** 2020-01-22

**Authors:** Shubhada R. Hegde

**Affiliations:** Institute of Bioinformatics and Applied Biotechnology, Bengaluru, India

**Keywords:** tuberculosis, biofilms, quorum sensing, protein interactions, evolution

## Abstract

With prolonged therapy and increased instances of drug resistance, tuberculosis is viewed as a serious infectious disease causing high mortality. Emerging concepts in *Mycobacterium tuberculosis* pathogenicity include biofilm formation, which endows bacterial survival in the host for a long time. To tackle chronic tuberculosis infection, a detailed understanding of the bacterial survival mechanisms is crucial. Using comparative genomics and literature mining, 115 *M. tuberculosis* proteins were shortlisted for their likely association with biofilm formation or quorum sensing. These include essential genes such as *secA2*, *lpqY-sugABC*, *Rv1176c*, and *Rv0195*, many of which are also known virulence factors. Furthermore, the functional relationship among these proteins was established by considering known protein-protein interactions, regulatory interactions, and gene expression correlation data/information. Graph centrality and motif analyses predicted the importance of proteins, such as Rv0081, DevR, RegX3, Rv0097, and Rv1996 in *M. tuberculosis* biofilm formation. Analysis of conservation across other biofilm-forming bacteria suggests that most of these genes are conserved in mycobacteria. As the processes, such as quorum sensing, leading to biofilm formation involve diverse pathways and interactions between proteins, these system-wide studies provide a novel perspective toward understanding mycobacterial persistence.

## Introduction

Tuberculosis claims millions of lives each year worldwide, thereby demanding immediate attention to discover efficient drug targets (Floyd et al., 2018). The foremost challenge in tuberculosis treatment is the emergence of persisters (Höner and Russell, 2001; Stewart et al., 2003). This makes antibiotic treatment less effective as the persister population is typically drug-resistant. So far, *Mycobacterium tuberculosis* persistence has been attributed to multiple mechanisms employed by the pathogen, such as residing in macrophages or bone marrow stem cells and/or forming biofilms (Höner and Russell, 2001; Ojha et al., 2008; Das et al., 2013). Notably, the persistence of most bacterial pathogens is facilitated by organized community structures called biofilms (Hall-Stoodley and Stoodley, 2009). Moreover, mycobacterial species, such as *M. marinum*, *M. fortuitum*, and *M. smegmatis*, have been shown to form biofilms (Bardouniotis et al., 2003; Ojha et al., 2005; Ojha and Hatfull, 2007). In *M. smegmatis*, cells in the biofilms showed decreased susceptibility to isoniazid treatment compared to planktonic cultures (Teng and Dick, 2003). This view is further strengthened by the roles of GroEL1 and iron-responsive genes in forming mature biofilms as observed in *M. smegmatis* (Ojha et al., 2005; Ojha and Hatfull, 2007).

In *Mycobacterium tuberculosis*, biofilms render increased resistance to drugs, such as isoniazid and rifampicin, *in vitro* (Ojha et al., 2008). Furthermore, a transposon insertional mutagenesis study in *M. tuberculosis* identified genes, such as *pks1*, a polyketide synthase, and *nirB*, which codes for a nitrite reductase, as important for biofilm formation and maturation (Pang et al., 2012). Recently, reductive stress was shown to induce biofilms in *M. tuberculosis*, which had cellulose as a key component of the extracellular matrix. Transcriptome analysis suggested that the bacteria in these biofilms were metabolically active while remaining drug-tolerant (Trivedi et al., 2016). Quorum-sensing systems enable the bacterial population to synchronize gene expression to achieve collective behaviors, such as biofilm formation (Ng and Bassler, 2009). Although detailed mechanisms of quorum sensing have not been reported so far for mycobacteria, proteins such as RelA, Rv1354c, and Rv1357c are associated with the metabolism of signaling molecules, which are potential regulators of community behaviors, such as biofilms (Gupta et al., 2010; Weiss and Stallings, 2013). However, compared to other biofilm-forming bacteria, very little is known about the proteins and their interactions, which lead to quorum sensing and biofilm formation in *M. tuberculosis*.

Since *M. tuberculosis* has the genetic capacity to form biofilms similar to other infectious bacteria, such as *Staphylococcus aureus* (SA), *Streptococcus pneumonia* (SM), and *Pseudomonas aeruginosa* (PA), the question arises whether it employs analogous mechanisms or has evolved with novel proteins and functions. Biofilm formation involves a spectrum of stages corresponding to initial attachment, growth, and detachment (Hall-Stoodley and Stoodley, 2009). Many transcription factors and the proteins from diverse pathways have been associated with bacterial biofilm formation (Ng and Bassler, 2009; Weiss and Stallings, 2013; Trivedi et al., 2016). Therefore, owing to its multifactorial orchestration, an ideal approach is to identify the key players using systems methods. In this study, *M. tuberculosis* biofilm and quorum sensing-associated proteins were identified using existing literature and comparative genomics, which were further supplemented with biomolecular interaction network and evolutionary analyses. These collective data provide the first systems-level description of proteins that could be potentially involved in *M. tuberculosis* community behavior, in turn enhancing mycobacterial survival and pathogenicity.

## Materials and Methods

### Identifying Quorum Sensing- and Biofilm-Associated Proteins

Proteins annotated with quorum sensing and biofilm were collected from the databases QuickGO^1^, NCBI-Protein, UniprotKB, and PDB (The UniProt Consortium, 2017; Haft et al., 2018; Burley et al., 2019). These protein sequences were derived from the NCBI database^2^ and clustered using CDHit with 90% sequence identity (Huang et al., 2010). In total, 5,323 biofilm-associated and 4,650 quorum sensing-associated bacterial proteins were compiled. This merged list of biofilm and quorum-sensing proteins was tested for their conservation in *M. tuberculosis* H37Rv. NCBI stand-alone BlastP with E-value cutoff 1e−04 was used for homology searches. To filter the blast hits, a cutoff of 70% coverage for both the query and the subject protein sequences, along with a bit score cutoff of 50, was used, followed by manual curation. All of the known quorum sensing- and biofilm-associated proteins in *M. tuberculosis* were derived using literature curation and manual inspection. Only the low-throughput experimentally validated genes were included in the final data. Protein functional categories were obtained from TubercuList database^3^. GO Molecular Function and Protein Classes were derived for the identified biofilm and quorum-sensing proteins using Panther database (Mi et al., 2017).

### Phylogenetic Conservation of the Biofilm- and Quorum Sensing-Associated Proteins (BQAPs)

Bacterial genome sequences and annotations were derived from the NCBI database (see text footnote 2), and the non-redundant database of these sequences was generated using CDHit with clusters of sequences with 90% identity (Huang et al., 2010). The representative sequences from each of these clusters were used for further analysis. For performing phylogenetic analysis, protein orthologs were identified using bidirectional BlastP with *E*-value cutoff of 1e−04. To filter the blast hits, a cutoff of 70% coverage for both the query and the subject protein sequences, and a bit score of 50, was used.

### Building Molecular Interaction Network

Genome-wide regulatory network for BQAPs was compiled from the given regulatory networks (Balázsi et al., 2008; Sanz et al., 2011; Minch et al., 2015). Genome-wide protein interaction network involving BQAPs was generated by merging the derived high-confidence protein interactions (Strong et al., 2003; Wang et al., 2010; Hegde et al., 2012; Liu et al., 2012; Szklarczyk et al., 2017; Wu et al., 2017). The gene expression correlations greater than 0.5 for BQAPs were obtained from Hegde et al. (2012). Network analysis, centrality measure calculation, and visualization were performed using Cytoscape (Shannon et al., 2003). R statistical tool was used to identify clusters and calculate other statistical measures^4^. *Z*-scores were calculated using 1,000 random networks with the same number of nodes. Network clusters were identified using CytoCluster application implemented in Cytoscape (Li et al., 2017). The identifying protein complex algorithm (IPCA) in CytoCluster was run with algorithm parameters set as Tin Threshold of 0.5 and shortest path length of 2.

## Results

### Identifying Quorum Sensing- and Biofilm-Associated Proteins in *Mycobacterium tuberculosis*

While genetic and biochemical studies have identified a few genetic regulators of biofilm formation in *M. tuberculosis*, such as pks1, nirB, Rv0199 (Pang et al., 2012), biofilm formation and quorum sensing remain underexplored areas of research. Since other bacterial pathogens adopt the biofilm mode of growth during latent infection, a comparative genomics approach was utilized to test if genes implicated in biofilm formation and quorum sensing in other pathogenic bacteria were conserved in *M. tuberculosis*.

By literature mining, 31 biofilm and four quorum sensing-associated proteins were identified in *M. tuberculosis* (Supplementary Table S1). In addition, seven proteins with a LuxR domain and two proteins associated with c-di-GMP metabolism were considered, as they are likely to participate in downstream signaling during quorum sensing (Gupta et al., 2010; Chen and Xie, 2011; Fang et al., 2013). Lastly, biofilm and quorum-sensing proteins that have been experimentally validated in other bacteria were gathered by querying multiple databases, and their homologs, if any, were detected in *M. tuberculosis* using BlastP (see section “Materials and Methods”). This resulted in the identification of 63 biofilm and nine quorum sensing-associated proteins. Protein RegX3 was identified as a LuxR domain protein, which was also detected in the homology search. Together, these 115 proteins are referred henceforth as BQAPs (Table 1 and Supplementary Table S1).

**TABLE 1:** Biofilm- and quorum sensing-associated proteins (BQAPs) categorized based on TubercuList functional classes.

**Functional categories**	**Count**	**Proteins**
Cell wall and cell processes	25	CeoB, Cut4, DacB2, DppA, EsxA, EsxB, FtsH, HbhA, LpqY, MgtE, MmpL11, MurB, MurE, Rfe, Tig, Rv0199, Rv0359, Rv1258c, Rv2136c, Rv3312A, SapM, SecA2, SugA, SugB, SugC
Conserved hypotheticals	14	Rv0021c, Rv0038, Rv0566c, Rv0574c, Rv1176c, Rv1354c, Rv1357c, Rv1991c, Rv1996, Rv2216, Rv2298, Rv2300c, Rv3237c, Rv3519
Information pathways	9	Fmt, HelY, Hns, Mfd, MutT3, NrdH, RplM, SigB, TypA
Intermediary metabolism and respiration	29	AckA, AroK, CarB, CcsA, CelA1, Cya, Eno, GalU, GltB, GuaB2, Icl, MoeW, MrsA, Mtn, MycP1, NirB, PmmB, Pnp, Ppk1, RelA, Rv0097, Rv0539, Rv1260, Rv2296, Rv2959c, Rv3526, Rv3784, Rv3790, Zwf2
Lipid metabolism	10	AccA2, AccD2, FabG4, InhA, KasA, KasB, Ltp3, Pks1, Pks10, Pks16
Regulatory proteins	22	DevR, FhaA, Lsr2, MprA, NarL, PknB, PknF, PknG, PknI, RegX3, Rv0081, Rv0195, Rv0386, Rv0890c, Rv0894, Rv1151c, Rv1358, Rv2488c, Rv2887, Rv3295, Rv3676, WhiB3
Virulence, detoxification, and adaptation	6	GroEL1, Rv0024, Rv1102c, Rv2801c, Rv3357, Rv3358

Analysis of GO molecular function and protein classes for the BQAPs showed the enrichment of protein kinases and transcription factors (Fisher’s exact with false discovery rate (FDR) < 0.05) (Table 2). These include serine/threonine protein kinases, such as PknB and PknG, and transcription factors, such as CRP, NarL, and RegX3. Also, the functional categories derived from TubercuList database show the enrichment of regulatory proteins (*P*-value < 7e−08). To test if the BQAPs constitute essential components for *M. tuberculosis* pathogenesis, these were compared with the available data on gene essentiality and virulence. Four studies were considered for generating *M. tuberculosis* gene essentiality data: (a) genomic regions derived from a saturating transposon mutagenesis screen (DeJesus et al., 2017); (b) genes required for *in vivo* survival during infection (Sassetti and Rubin, 2003); (c) genes required for survival in primary macrophages (Rengarajan et al., 2005); and (d) genes essential for *in vivo* survival in primates (Dutta et al., 2010). The virulence genes of *M. tuberculosis* were obtained (Kalscheuer et al., 2010; Forrellad et al., 2013; Garg et al., 2015). Of the 115 BQAPs, 38 are found to be essential for growth (*P*-value < 0.02), and 23 are reported to be virulence factors (*P*-value < 7.5e−09). Also, 18 genes are required for growth *in vivo* (*P*-value < 0.05), suggesting that BQAPs are overrepresented for the essential genes and the virulence factors (Table 3). Following are some of the essential BQAPs that are discussed in detail.

**TABLE 2:** Enriched functional classes of the identified biofilm- and quorum sensing-associated proteins (BQAPs).

**Functional category**	**Over (+)/ Under (−)**	**Fold enrichment**	**FDR**
**GO molecular function**			
DNA binding (GO:0003677)	+	2.43	2.78E−02
Protein binding (GO:0005515)	+	2.34	4.92E−02
Heterocyclic compound binding (GO:1901363)	+	1.81	9.06E−04
Organic cyclic compound binding (GO:0097159)	+	1.81	7.25E−04
Binding (GO:0005488)	+	1.63	1.33E−04
Molecular function (GO:0003674)	+	1.43	1.32E−07
Unclassified	−	0.28	2.65E−07
**PANTHER protein class**			
Protein kinase (PC00193)	+	7.43	1.04E−02
Kinase (PC00137)	+	4.78	1.09E−02

**TABLE 3:** List of biofilm- and quorum sensing-associated proteins (BQAPs) implicated in essentiality and/or virulence.

**Genes**	**A**	**B**	**C**	**D**	**E**
*secA2*, *lpqY*, *sugA*, *sugB*, *sugC*	0	0	1	1	1
*Rv0566c*, *pks16*, *Rv3519*, *ltp3*	0	0	0	1	0
*Rv0195*, *ceoB*	0	0	1	0	0
*Rv1176c*, *dacB2*	0	1	0	0	0
*Rv0199*	0	0	0	1	1
*regX3*	0	1	0	0	1
*fhaA*	1	0	1	0	0
*icl1*	1	0	0	0	1
*Rv0574c*, *mmpL11*, *pknG*, *hbhA*, *mprA*, *pks10*, *Rv2136c*, *kasB*, *pks1*, *devR*, *whiB3*, *dppA*, *esxB*, *esxA*, *mycP1*	0	0	0	0	1
*pknB*, *murB*, *ccsA*, *galU*, *eno*, *rfe*, *carB*, *fmt*, *inhA*, *murE*, *kasA*, *aroK*, *ppk1*, *nrdH*, *guaB2*, *mrsA*, *rplM*, *lsr2*, *dprE1*, *gltB*	1	0	0	0	0

*(A) Saturating transposon mutagenesis study (DeJesus et al., 2017). (B) Genes essential for survival in primates (Dutta et al., 2010). (C) Genes required for survival in macrophages (Rengarajan et al., 2005). (D) Genes required for mycobacterial survival *in vivo* (Sassetti and Rubin, 2003). (E) Virulence factors of the Mycobacterium tuberculosis complex (Forrellad et al., 2013). In the represented binary matrix format, 1 represents virulent or an essential gene.*

Free mycolic acids (FM) form one of the major components of the *M. tuberculosis* biofilm matrices (Ojha et al., 2008). In mycobacteria, trehalose acts as a conjugate to transport mycolic acids outside the cell in the form of a trehalose monomycolate (TMM). This extracellular trehalose moiety is recycled into the cell by the ABC transporter LpqY-SugABC. The genes *lpqY*, *sugA*, *subB*, and *sugC* are predicted to be associated with the biofilm formation by comparative genomic analysis (Supplementary Table S1). These act as virulence factors, which are also required for *in vivo* growth (Sassetti and Rubin, 2003; Rengarajan et al., 2005; Kalscheuer et al., 2010). In *M. smegmatis*, the anti-biofilm activity of the trehalose analogs was dependent on their uptake by LpqY-SugABC, suggesting that the import of trehalose by LpqY-SugABC was critical for *M. smegmatis* biofilm formation (Wolber et al., 2017). The uptake of trehalose is also shown to be important for *M. tuberculosis* virulence during infection (Kalscheuer et al., 2010). These genes are also shown to be required for both *in vivo* and macrophage growth (Sassetti and Rubin, 2003; Rengarajan et al., 2005). As the continual release of the FM to the biofilm matrix depends on trehalose recycling, the transporter LpqY-SugABC is likely to play an important role in *M. tuberculosis* biofilm formation.

SecA2 is a predicted biofilm-associated protein, which is required for *in vivo* and macrophage growth (Sassetti and Rubin, 2003; Rengarajan et al., 2005). In *M. avium*, *secA* expression correlates with biofilm formation (Limia et al., 2001). Expression of *secA* was increased during biofilm growth in *Streptococcus mutans* (SM) (Huang et al., 2008). The secretory role of SecA2 might be important for the biofilm formation as it exports a subset of proteins, which also promote *M. tuberculosis* virulence (Miller et al., 2017).

Two other BQAPs, Rv0199, and Rv3523, are essential for *in vivo* survival during infection (Sassetti and Rubin, 2003). Transposon mutagenesis screen in *M. tuberculosis* identified Rv0199 as one of the proteins required for biofilm growth (Pang et al., 2012). Rv0199 is shown to be one of the exported proteins, the mutant of which shows attenuated growth in macrophages (McCann et al., 2011). Rv3523, which is a lipid carrier protein. Ltp3 is upregulated in *M. avium* biofilms (Yamazaki et al., 2006). LuxR transcriptional regulators play a key role in quorum sensing by regulating the expression of genes involved in the processes such as virulence, motility, and biofilm formation (Chen and Xie, 2011). Rv0195 is a LuxR family regulator, which is required for the survival in murine macrophages (Rengarajan et al., 2005). Deletion of Rv0195 showed attenuated virulence in human THP-1 cells and mouse tissues (Fang et al., 2013). Therefore, studying these proteins in the context of biofilm and quorum-sensing mechanisms could be important for explaining their *in vivo* essentiality.

### Conservation of Biofilm- and Quorum Sensing-Associated Proteins Across Biofilm-Forming Bacteria

**FIGURE 1:**
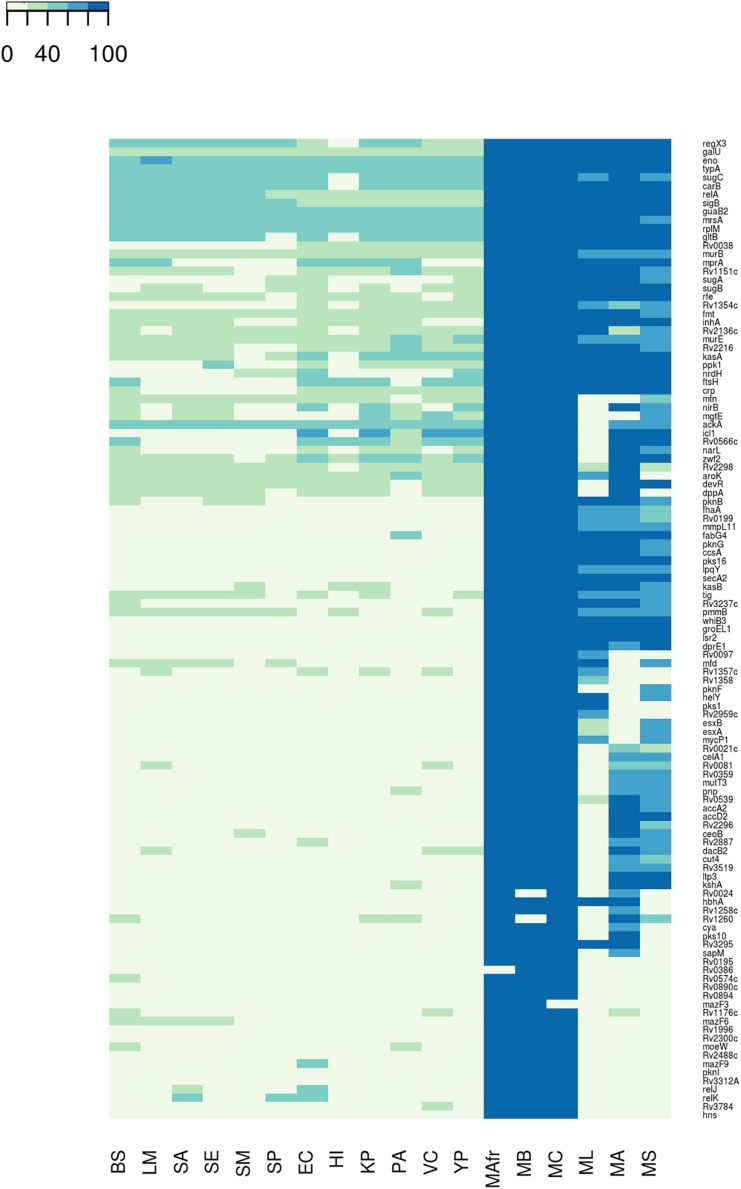
Conservation of the biofilm- and quorum sensing-associated proteins (BQAPs) across bacterial genomes. Percent identities between the homologs are used for generating the heat map.

We next asked whether the BQAPs identified here are conserved across all biofilm-forming bacteria or are specific to mycobacteria. With the availability of many genome sequences, phylogenetic profiling is a useful method to identify conserved genes across species (Enault et al., 2003). For the 115 BQAPs, which were derived using literature curation and comparative genomics, phylogenetic study was performed to assess their conservation across mycobacteria and some of the other widely studied biofilm-forming bacteria. The following genomes were considered for the phylogenetic analysis: Gram negative bacteria – *Escherichia coli* (EC), *Haemophilus influenzae* (HI), *Klebsiella pneumoniae* (KP), *P. aeruginosa* (PA), *Vibrio cholerae* (VC), and *Yersinia pestis* (YP); Gram positive bacteria – *Bacillus subtilis* (BS), *Listeria monocytogenes* (LM), *S. aureus* (SA), *Staphylococcus epidermidis* (SE), *S. mutans* (SM), and *S. pneumoniae* (SP). Also, the following mycobacterial genomes were included: pathogenic mycobacteria from the *Mycobacterium tuberculosis* complex (TP-MTBC) – *Mycobacterium africanum* (MAfr), *Mycobacterium canetti* (MC), *Mycobacterium bovis* (MB), opportunistic pathogen *M. avium* (MA), which is a non-tuberculous mycobacteria (NTM), non-pathogenic mycobacteria *Mycolicibacterium smegmatis*, basonym *Mycobacterium smegmatis* (MS), and *M. leprae* (ML), which is an obligate intracellular pathogenic mycobacterium with extensive gene reduction. For each of the BQAPs, presence of the homologs was tested across these genomes using BlastP (see section “Materials and Methods”). Percent identities of the identified homologs were used for further analysis (Figure 1 and Supplementary Table S2).

**FIGURE 2:**
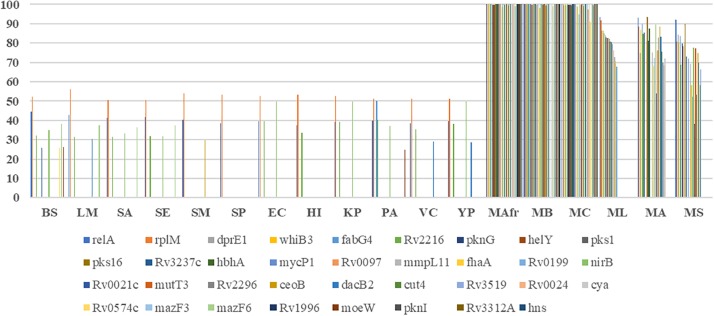
Conservation of the curated biofilm and quorum sensing-associated genes. *X*-axis refers to the genome, and *Y*-axis represents the percent identity between the identified homologs, if detected.

Most of the literature curated genes were specific to mycobacteria. In the curated set of 35 genes, only *relA*, *rplM*, *Rv2216*, and *nirB* are conserved across bacterial genomes, while the remaining 31 genes are largely seen in mycobacteria (Figure 2). Within mycobacteria, only 12 curated genes are highly conserved in all of the studied pathogenic and non-pathogenic mycobacterial genomes. While *pks1* and *Rv0097* are present only in the TP-MTBC genomes, gene *hbhA* is present in all of the studied mycobacterial genomes except a non-pathogenic *M. smegmatis. M. leprae* seems to have lost many reported biofilm-associated genes, as the genes such as *Rv0021*, *Rv2296*, *nirB*, and *Rv3519* are selectively absent in *M. leprae* compared to the other mycobacterial genomes. Also, *Rv0574c*, *mazF3*, *mazF6*, *Rv1996*, *moeW*, *pknI*, *Rv3312A*, and *hns* are exclusively present in TP-MTBC genomes excluding *M. leprae.* The loss of multiple biofilm- and quorum sensing-associated genes might explain the reduced virulence of *M. leprae*.

A similar trend is seen for the predicted biofilm-associated proteins. There are 10 genes that are highly conserved across bacterial genomes, for example, *sigB*, *typA*, *relA*, and *mrsA*. About 28 genes, including *Rv1151c* and *Rv2136c*, are moderately conserved in other genomes but well-conserved within mycobacteria. Also, genes such as *Rv0038*, *Rv1354c*, and *Rv0467* are selectively present in mycobacteria and Gram-negative bacteria. The homologs of *Rv0566c*, *narL*, *ftsH*, *crp*, and *mprA* are absent in the Gram-positive bacteria, except *B. subtilis*. These examples highlight the species-specific proteins involved in the bacterial biofilm formation.

Within mycobacteria, all of the BQAPs are highly conserved among TP-MTBC members, namely, *M. africanum*, *M. canetti*, and *M. bovis.* Of the 80 predicted BQAPs, only 38 are conserved in all of the studied mycobacteria. The genes *Rv3295* and *Rv2539c* are selectively absent in a non-pathogenic *M. smegmatis* compared to the other mycobacteria. Also, *Rv2959c*, *Rv1357c*, and *Rv1358* are exclusively present in TP MTBC genomes. Similar to curated genes, many predicted BQAPs are absent in *M. leprae.* Supplementary Table S2 shows the percent identities of the homologs of BQAPs in all of the studied genomes and their annotated functions. Therefore, in addition to the genes that are conserved across most of the biofilm-forming bacteria, we find genes that seem to be specific to mycobacteria, suggesting that mycobacteria probably have evolved with some unique mechanisms of biofilm formation and regulation.

### Interaction Network Reveals Functional Connectedness Among Biofilm- and Quorum Sensing-Associated Proteins

**FIGURE 3:**
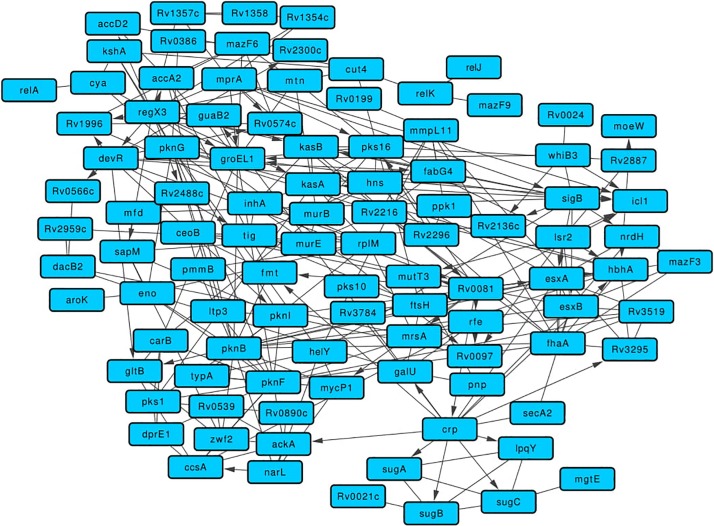
Merged network comprising of protein-protein, gene regulatory, and expression correlation interactions for the BQAPs.

The 115 BQAPs identified using literature curation and comparative genomic analyses belong to diverse pathways and functional classes with enrichment for kinases and the transcription factors (Supplementary Tables S1, S2). To understand their functional association, a network approach involving graph theoretical analyses was performed. A merged network of protein-protein interactions, expression correlations, and gene regulatory interactions was derived for the identified BQAPs. The resulting network had 275 interactions among 101 proteins, which formed a single connected component (Figure 3 and Supplementary Table S3). This network was densely connected, suggesting an underlying functional relation between BQAPs (*Z*-score: 2.74; *P*-value < 0.003). To shortlist important proteins that connect these proteins, network centrality measures, namely, degree, betweenness, closeness, and stress centrality, were calculated (see section “Materials and Methods”). There are 37 proteins that appear in the top 25% of these centrality measures, of which 22 genes are shown to be essential and/or virulence associated (Figure 3 and Supplementary Table S4).

One of the highly connected transcription regulators is Rv0081, which is homologous to the protein BigR of *Xylella fastidiosa*. BigR is a transcriptional repressor with a helix-turn-helix motif, the mutant of which showed increased biofilm formation in *X. fastidiosa* (Barbosa and Benedetti, 2007). In the network, Rv0081 regulates 13 genes, including five curated biofilm-associated proteins, Pks16, MycP1, RenU, Rv0097, and HbhA (Supplementary Figure S1). Similarly, protein DevR is homologous to LiaR of *S. mutans*, *S. uberis*, *and B. subtilis*. In *S. mutans*, deletion of *hk11* and *rr11*, which correspond to LiaS and LiaR, respectively, resulted in the defective biofilms (Li et al., 2002). On the other hand, transposon insertional mutagenesis of LiaR in *S. uberis* resulted in increased biofilm production (Salomäki et al., 2015). In the network, DevR regulates *fabG4*, *pknI*, *Rv0574c*, and *Rv1996* (Supplementary Figure S1). It is interesting to note that both *Rv0081* and *devR* are downregulated in the *M. tuberculosis* biofilms (Trivedi et al., 2016).

Another BQAP with high centrality is RegX3, a two-component response regulator that belongs to the LuxR family. This protein is essential for the *in vivo* bacterial growth and required for virulence (Parish et al., 2003; Dutta et al., 2010). RegX3 is homologous to WalR of *S. aureus*, which is a positive regulator of peptidoglycan biosynthesis and biofilm formation (Dubrac et al., 2007). RegX3 regulates the expression of 10 other BQAPs, including two curated genes *Rv0097* and *Rv1996* (Parish et al., 2003; Supplementary Figure S1). *M. tuberculosis* Lsr2, which has a high centrality in the network, regulates the expression of the curated BQAPs, such as HbhA, MutT3, and Rv0097 (Supplementary Figure S1). In *M. smegmatis*, *lsr2* transposon insertion mutant showed altered colony morphology and defective biofilm formation. This mutant lacked apolar lipids called mycolyl-diacylglycerols (MDAGs) in the cell wall (Chen et al., 2006). In mycobacterium biofilms, *lsr2* expression is upregulated (Trivedi et al., 2016). Therefore, it is plausible that Lsr2 is involved in regulating cell wall morphology, thereby contributing to biofilm formation in *M. tuberculosis*.

One of the highly connected regulators identified in the quorum-sensing category is MprA, which regulates seven genes, including two curated genes *pks16* and *Rv0574c* (Supplementary Figure S1). MprA is a response regulator of the two-component system MprA/MprB, which is also implicated in virulence (Forrellad et al., 2013). MprA is homologous to QseB of *H. influenzae*, which is implicated in quorum sensing and biofilm formation (Unal et al., 2012).

Some of the high centrality proteins such as Rv0020c, Rv0097, and Rv1996 are pellicle-specific proteins determined by protein expression (Kerns et al., 2014). Rv0020c is required for both *in vitro* growth and survival in macrophages (Rengarajan et al., 2005; DeJesus et al., 2017). Two other proteins, namely, CcsA and GalU, are essential for *in vitro* growth (DeJesus et al., 2017). CcsA, which is a putative cytochrome C-type biogenesis protein, is upregulated during biofilm formation in *M. avium* (Yamazaki et al., 2006).

Some of the known virulence factors appear as high centrality nodes in the network. Mutation in *pks1* results in biofilm maturation deficiency (Pang et al., 2012). Protein HbhA, which is a heparin-binding hemagglutinin, helps in mycobacterial aggregation and acts as an adhesin by binding to epithelial cells (Menozzi et al., 1996). HbhA also functionally interacts with other known biofilm-associated proteins, such as PknG, PknI, FhaA, and Rv2216 (Figure 3). MmpL11 is a transmembrane lipid transport protein required for *M. tuberculosis* pathogenicity. Mutants of *mmpL11* had altered biofilms and showed impaired growth in an *in vitro* human granuloma model (Wright et al., 2017).

### Highly Inter-Connected Biofilm- and Quorum Sensing-Associated Proteins Deduced by Identifying Network Motifs

While centrality measures capture the proteins that are important for maintaining the global structure of the network, network clusters are densely connected groups of nodes with related functions. The substructures within these clusters are the motifs (or subgraphs) that show underlying local connections between proteins (Milo et al., 2002). To identify such local associations between curated and predicted BQAPs, the molecular interaction network was split into densely connected clusters of proteins (see section “Materials and Methods”). We identified 37 such clusters with a size of at least four proteins (Supplementary Table S5). Of these, there were 27 clusters that had at least one curated protein.

**FIGURE 4:**
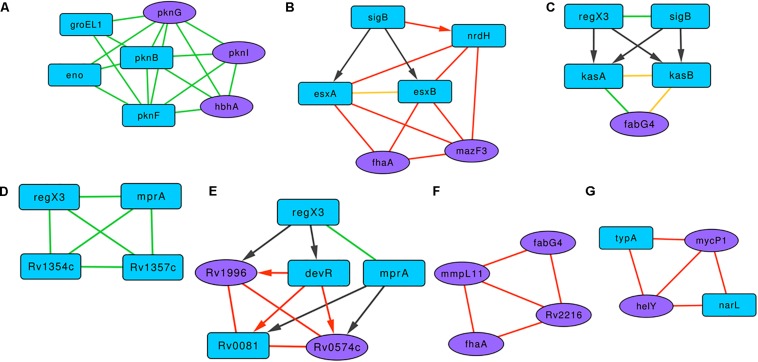
Representative motifs enriched in the Molecular Interaction Network of the BQAPs. **(A)** A subgraph comprising of protein interactions between serine/threonine protein kinases (STPKs), HbhA, Eno and GroEL1. **(B)** A subgraph with regulatory interactions of SigB and other proteins with high expression correlations. **(C)** A bifan motif comprising of RegX3 and SigB. **(D)** A fully connected protein interaction tetrad between RegX2, MprA, Rv1354c and Rv1357c. **(E)** A subgraph involving regulatory proteins DevR, Rv0081, MprA and RegX3, and subgraphs showing high expression correlations **(F)** between MmpL11, FabG4, FhaA and Rv2216, and **(G)** between MycP1, HelY, NarL and TypA. Blue nodes- predicted proteins, purple nodes - curated proteins, green edges - protein interactions, black edges - gene regulation, red edges - high gene expression correlation, yellow edges - interacting proteins with high gene expression correlation, undirected edge - protein interaction or high gene expression correlation, directed edge - gene regulation.

A highly clustered subgraph of protein-protein interactions revealed interactions between serine/threonine protein kinases (STPKs) and the proteins HbhA, Eno, and GroEL1 (Figure 4A). In *M. smegmatis*, the overexpression of *pknF* resulted in the defective biofilm formation, possibly affecting the GPL metabolism (Gopalaswamy et al., 2008). In this subgraph, PknF shows protein interactions with the three of the known biofilm-associated proteins PknG, PknI, and HbhA, suggesting its possible role in regulating *M. tuberculosis* biofilms. In the subgraph Figure 4B with six proteins and 12 interactions, RNA polymerase sigma factor SigB regulates the expression of *esxA*, *esxB*, and *nrdH*. These three proteins show a connected web of expression correlations among themselves along with two curated proteins FhaA and MazF3.

There are nine clusters that involve the response regulator RegX3. One of them is a subgraph with RegX3, SigB, KasA, KasB, and FabG4 (Figure 4C). RegX3 and SigB form a bifan motif to regulate the expression of lipid metabolism proteins KasA and KasB, and these interact with FabG4, which is a biofilm-specific protein (Garg et al., 2015). Proteins KasA, KasB, and FabG4 are connected by both protein-protein and expression correlation interactions, forming a fully connected tetrad. In another subgraph involving RegX3, proteins MprA, Rv1354c, and Rv1357c form a fully connected tetrad of protein interactions (Figure 4D). All of the members of this triad are associated with quorum sensing. Cyclic di-GMP (c-di-GMP) is one of the important signaling molecules in the cell, and the role of c-di-GMP in the biofilm progression is studied in many bacteria, including *P. aeruginosa* (Valentini and Filloux, 2016). Therefore, the interactions between c-di-GMP metabolism proteins Rv1354c and Rv1357c with the regulators RegX3 and MprA might be important for *M. tuberculosis* biofilm progression.

Another subgraph marked the interactions between transcription regulators DevR, Rv0081, MprA, and RegX3 and the biofilm-associated proteins Rv1996 and Rv0574c (Figure 4E). Proteins Rv0081, DevR, Rv0574c, and Rv1996 form a fully connected tetrad of expression correlations. Within this subgraph, there is a bifan motif, which involves DevR and MprA, both regulating Rv0081 and Rv0574c. Moreover, a feed-forward loop connects RegX3, DevR, and Rv1996. Of these, three genes, *Rv0081*, *Rv1996*, and *devR*, are reported to be downregulated in *M. tuberculosis* biofilms (Trivedi et al., 2016).

There are subgraphs that show connections based on the expression correlations alone. Figure 4F is an expression correlation clique involving five edges between the proteins MmpL11, FabG4, FhaA, and Rv2216. Of these FhaA, FabG4, and Rv2216 are identified as antigenic pellicle proteins (Kerns et al., 2014). Also, MmpL11 is a virulent factor, the mutant of which has altered biofilms (Wright et al., 2017). Another expression correlation-based subgraph constitutes two curated proteins, MycP1 and HelY, both interacting with predicted proteins NarL and TypA (Figure 4G). NarL is homologous to DegU of *B. subtilis*, which acts as a positive regulator of biofilm formation (Murray et al., 2009). It is therefore likely that these clusters of interactions play a significant role in *M. tuberculosis* biofilm formation.

## Discussion

While tuberculosis remains one of the deadliest infectious diseases, a detailed understanding of how it persists in the host is still not complete. One of the mechanisms by which pathogenic bacteria achieve increased persistence is by forming self-organized community structures termed biofilms. The importance of such pellicle biofilms in mycobacterial pathogenesis is noted in a study that demonstrates biofilms imparting drug resistance to an otherwise drug-sensitive strain of *M. tuberculosis* (Ojha et al., 2008). Since biofilm formation is a complex behavior involving diverse pathways, system-wide studies are required to understand it better. Through comparative genomic analyses and literature mining, 115 proteins were identified to be associated with biofilm and/or quorum sensing in *M. tuberculosis*. Also, there are additional proteins, such as PpiB, resuscitation promoting factors (rpf), and GlmU, which have been recently reported in association with the mycobacterial biofilms (Ealand et al., 2018; Di Somma et al., 2019; Kumar et al., 2019). Mutation of resuscitation promoting factor (rpf) genes showed altered colony morphology and impaired biofilm formation in *M. smegmatis* (Ealand et al., 2018). Similarly, depletion of GlmU in the early stages of *M. smegmatis* growth showed a decrease in the biofilm production (Di Somma et al., 2019). Collectively, these genes could be the potential candidates for further biofilm-related studies in mycobacteria.

Furthermore, these proteins show enrichment for essential genes and virulence factors, suggesting their role in mycobacterial pathogenicity. Their connectedness was established using gene regulatory interactions, protein-protein interactions, and gene expression correlations. Collectively, these molecular interactions represented as a network provide wider perspectives on how proteins are linked in terms of their function. Proteins such as Rv0081, RegX3, DevR, and MprA are some of the high centrality nodes in the network that are also the members in many of the enriched motifs involving known biofilm-associated proteins. These could be of interest for further experimental validation for their association with biofilm and quorum sensing.

Gene conservation analysis across bacterial species along with six mycobacterial genomes suggested that there are probably mycobacterial specific mechanisms governing biofilm formation and regulation in addition to the ones that are conserved in some of the other biofilm-forming bacteria. Of the 115 BQAPs, only 42 genes including *mrsA*, *relA*, and *sigB* show homologs in most of the studied bacterial genomes, and 33 genes are absent in the non-pathogenic mycobacteria *M. smegmatis*. Similarly, 31 proteins including 12 literature curated proteins are absent in an opportunistic pathogen *M. avium*. Overall, BQAPs are highly conserved among TP-MTBC genomes, such as *M. africanum*, *M. canetti* and *M. bovis.* However, there is a significant loss of BQAPs in *M. leprae*, with 52 genes being absent in *M. leprae*, including 18 curated proteins. Proteins reported to be associated with biofilm formation, such as Rv1996 and Rv3312A, are uniquely present in MTB complex. Hypothetical protein Rv0021c, the mutant of which is attenuated for biofilm formation, is absent in *M. leprae* but conserved in the other MTBC, *M. avium*, *M. smegmatis*, and Gram-positive bacteria. This protein also shows high expression correlation with SugB, which is an essential gene predicted to be associated with biofilm formation. Therefore, the selective conservation of biofilm and quorum sensing-associated genes sheds light on the evolution of mycobacterial pathogenicity.

## Data Availability Statement

All datasets generated for this study are included in the article/Supplementary Material.

## Author Contributions

SH conceived the study, carried out the analysis, and wrote the manuscript.

## Conflict of Interest

The authors declare that the research was conducted in the absence of any commercial or financial relationships that could be construed as a potential conflict of interest.

## Footnotes

1 https://www.ebi.ac.uk/QuickGO/

2 https://www.ncbi.nlm.nih.gov/

3 http://genolist.pasteur.fr/TubercuList/

4 https://www.r-project.org/

## Supplementary Material

FIGURE S1 Gene regulatory network depicting interactions between transcription factors (blue) and their target genes (green) among BQAPs.

TABLE S1 List of 115 BQAPs with annotations.

TABLE S2 Percent identities of the conserved BQAP homologs across biofilm forming bacteria.

TABLE S3 Molecular interaction network of the BQAPs.

TABLE S4 List of high centrality nodes in the BQAP molecular interaction network.

TABLE S5 Interactions between nodes in the identified network motifs.
