# Supplementary figures and images for: Computational Identification of the Proteins Associated With Quorum Sensing and Biofilm Formation in Mycobacterium tuberculosis
Source: Front Microbiol. 2020 Jan 22;10:3011. doi: 10.3389/fmicb.2019.03011 (PMC6988586; doi:10.3389/fmicb.2019.03011)

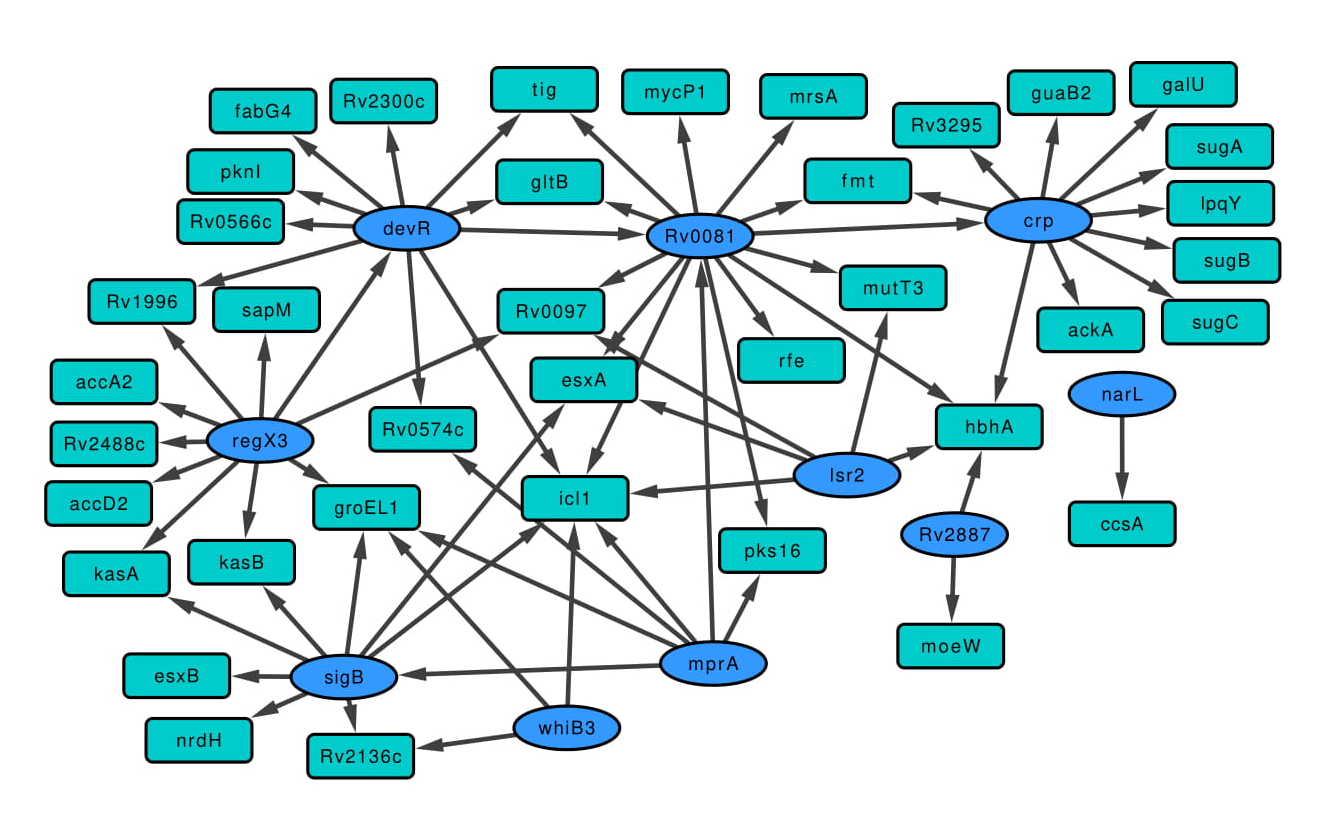

Supplement: FIGURE S1 — Gene regulatory network depicting interactions between transcription factors (blue) and their target genes (green) among BQAPs. [file Image_1.TIF]
